# Anesthesia for Minimal Invasive Cardiac Surgery: The Bonn Heart Center Protocol

**DOI:** 10.3390/jcm13133939

**Published:** 2024-07-05

**Authors:** Florian Piekarski, Marc Rohner, Nadejda Monsefi, Farhad Bakhtiary, Markus Velten

**Affiliations:** 1 Department of Anesthesiology and Intensive Care Medicine, Rheinische Friedrich-Wilhelms-University, University Hospital Bonn, 53127 Bonn, Germany; marc.rohner@ukbonn.de (M.R.); markus.velten@utsouthwestern.edu (M.V.); 2 Department of Cardiac Surgery, Rheinische Friedrich-Wilhelms-University, University Hospital Bonn, 53127 Bonn, Germany; nadejda.monsefi@ukbonn.de (N.M.); farhad.bakhtiary@ukbonn.de (F.B.); 3 Department of Anesthesiology and Pain Management, Division of Cardiovascular and Thoracic Anesthesiology, University of Texas Southwestern Medical Center, Dallas, TX 75390, USA

**Keywords:** anesthesia, minimally invasive cardiac surgery, management

## Abstract

The development and adoption of minimally invasive techniques has revolutionized various surgical disciplines and has also been introduced into cardiac surgery, offering patients less invasive options with reduced trauma and faster recovery time compared to traditional open-heart procedures with sternotomy. This article provides a comprehensive overview of the anesthesiologic management for minimally invasive cardiac surgery (MICS), focusing on preoperative assessment, intraoperative anesthesia techniques, and postoperative care protocols. Anesthesia induction and airway management strategies are tailored to each patient’s needs, with meticulous attention to maintaining hemodynamic stability and ensuring adequate ventilation. Intraoperative monitoring, including transesophageal echocardiography (TEE), processed EEG monitoring, and near-infrared spectroscopy (NIRS), facilitates real-time assessment of cardiac and cerebral perfusion, as well as function, optimizing patient safety and improving outcomes. The peripheral cannulation techniques for cardiopulmonary bypass (CPB) initiation are described, highlighting the importance of cannula placement to minimize tissue as well as vessel trauma and optimize perfusion. This article also discusses specific MICS procedures, detailing anesthetic considerations and surgical techniques. The perioperative care of patients undergoing MICS requires a multidisciplinary approach including surgeons, perfusionists, and anesthesiologists adhering to standardized treatment protocols and pathways. By leveraging advanced monitoring techniques and tailored anesthetic protocols, clinicians can optimize patient outcomes and promote early extubation and enhanced recovery.

## 1. Introduction

The development and implementation of minimally invasive techniques have significantly advanced various surgical specialties, including cardiac surgery. Minimal invasive cardiac surgery (MICS) offers patients less invasive approaches, resulting in reduced trauma and faster recovery time compared to traditional open-heart surgeries requiring sternotomy and its associated consequences, such as blood loss, infection, or increased postoperative pain [1,2,3]. MICS employs small incisions, specialized instruments, and advanced imaging technologies, enabling surgeons to perform intricate procedures with enhanced precision and minimal disruption to surrounding tissues. This reduces blood loss and, most notably, avoids sternotomies and their associated consequences [4].

The American Heart Association (AHA) defines MICS as surgical procedures performed through small chest wall incisions, typically thoracotomy, as opposed to traditional cardiac surgical procedures using sternotomy [5]. Consequently, catheter-based, transvascular approaches for structural heart diseases are not included in this scope. In addition to better cosmetic results [1], the advantages of MICS include reduced postoperative pain, a shorter hospital stay, and a lower risk of wound infection, bleeding, respiratory complications, and atrial fibrillation [2,3].

This approach has led to shorter duration of intensive care treatments, reduced hospital length of stay, and decreased incidence of hemodilution, bleeding, and the need for red blood cell (RBC) transfusion [6]. Furthermore, improvements in hematocrit, decreased post-operative pain, and a faster return to normal activities have been reported after MICS [7]. These findings mark a profound shift in perioperative cardiosurgical care. The number of MICS procedures continues to grow, encompassing a range of surgical techniques including bypass, valve, and aortic surgery, performed with the utilization or in the absence of cardiopulmonary bypass (CPB). A common feature of all MICS procedures is their performance in the absence of a sternotomy [4]. The expansive range of MICS includes minimally invasive direct coronary artery bypass (MIDCAB), valve repair and replacement, repair of septal defects, aortic procedures, and pulmonary vein ablation for atrial fibrillation [4,8].

The management of patients undergoing minimally invasive cardiac surgery requires not just detailed knowledge and experience regarding the corresponding procedure on the part of the surgeon, but also depends on a highly specialized cardio-anesthesiology team and established interdisciplinary treatment pathways.

At the Bonn Heart Center, a significant number of procedures are performed utilizing minimally invasive cardiac surgery (MICS) techniques. The presented recommendations are based on the current international literature and our institutional experience.

This article presents the anesthetic management for MICS on the basis of the Bonn Heart Center protocol and provides institutional treatment recommendations.

## 2. Preanesthetic Assessment

The objective of the pre-anesthesia assessment includes the assessment and evaluation of the patient’s vascular access required for peripheral bypass cannulation. This varies depending on the corresponding procedure and anatomical variations, as well as relevant pre-existing conditions, including cardiac function, coronary artery disease, and pulmonary function [9]. An adequate risk stratification is carried out in order to optimize the patient’s preoperative conditions. For planned surgeries, patients are evaluated by a cardiothoracic anesthetist at least one day prior to the procedure to identify potential contraindications and individual considerations. Some MICS procedures, such as minimally invasive direct coronary artery bypass (MIDCAB) surgery, require one-lung ventilation (OLV). Consequently, lung function tests and blood gas analysis are conducted, and the perioperative management is adapted accordingly.

It is essential to evaluate the presence of pulmonary hypertension and right ventricular dysfunction, as the utilization of OLV may potentially pose risks to these patients. Complications such as elevated pulmonary arterial pressure, heightened right ventricular afterload, and cardiac failure may arise as a result of OLV in this patient population [10].

Transesophageal echocardiography is of the highest importance for the echocardiographic guidance of minimally invasive cardiac procedures. Therefore, conditions of the upper gastrointestinal tract, in particular documented esophageal diseases, hiatal hernias, or previous operations and strictures, require evaluation. MICS is relatively contraindicated in cases of revision surgery, low cardiac output syndrome, or a severely reduced ejection fraction. The administration of sedative premedication is typically not included as part of the ERACS concept.

## 3. Induction

Following the implementation of standard hemodynamic monitoring, including electrocardiography (ECG) and pulse oximetry (SpO2), the left radial artery is cannulated under local anesthesia and ultrasound guidance. Subsequently, invasive blood pressure monitoring is conducted, and anesthesia is induced following preoxygenation with an opioid, such as remifentanil or sufentanil, a hypnotic agent (propofol), and a muscle relaxant (rocuronium). Anesthesia is maintained throughout the CPB procedure using volatile anesthetics, unless there is a suspicion of susceptibility to malignant hyperthermia or mitochondriopathy.

## 4. Airway Management

Following anesthesia induction and paralysis, endotracheal intubation is performed in accordance with a standardized institutional protocol, utilizing a single lumen tube for the majority of procedures. This is contingent upon a thorough assessment of the airway. The decision to employ video laryngoscopes is based on the results of the preoperative airway evaluation. The deflation of different lung parts is required, dependent on the performed MICS procedure. Various publications suggest lung isolation using a double-lumen endobronchial tube (DLT) or the utilization of a bronchus blocker. A prolonged duration of induction and total operative time has been observed in patients undergoing lung isolation by DLT, with no difference in the duration of ICU stay [11]. We utilize a single lumen tube for all on-pump MICS procedures and deflate both lungs after CPB has been established. Lung isolation using a left-sided DLT is established only for off-pump MIDCAB procedures. In this case, a left-sided DLT is the preferred option since right-sided DLTs have shown to be associated with poorer clinical performance [12]. Alternatively, in the event of a difficult airway, a single lumen tube is placed and then OLV is applied via bronchus blocker isolation of the lung. Lung deflation during MICS is associated with the potential occurrence of a pulmonary re-expansion edema, a rare but potentially catastrophic complication that may arise when the collapsed lung re-expands rapidly post-operation [13]. The sudden expansion can strain the pulmonary vasculature, leading to increased capillary permeability and fluid leakage into the interstitial spaces of the lung parenchyma. Consequently, patients may present with symptoms such as dyspnea, cough, and hypoxemia. It is important to understand the risk factors, including long CPB durations, diabetes, chronic obstructive pulmonary disease, right ventricular dysfunction, high pulmonary artery pressure, intraoperative fresh frozen plasma transfusion, and a high perioperative C-reactive protein level, and to implement preventive strategies during perioperative care [14]. These strategies play a pivotal role in mitigating the occurrence of pulmonary re-expansion edema, thereby optimizing patient outcomes following minimal invasive cardiac surgery. The use of total deflation has not resulted in an increase in the number of cases of clinically significant pulmonary edema that required invasive treatment.

## 5. Intraoperative Management

### 5.1. Monitoring

Irrespective of the surgical approach (minimally invasive or conventional), patients receive comprehensive cardio-anesthesiologic monitoring. Basic monitoring includes a five-channel ECG with ST segment analysis, SpO_2_, etCO_2_, temperature, invasive arterial blood pressure, central venous pressure, cerebral oximetry using near-infrared spectroscopy (NIRS), processed EEG (Bispectral Index (BIS)), and a comprehensive TEE evaluation. A pulmonary artery catheter is utilized in patients with pulmonary artery hypertension or right ventricular dysfunction. External defibrillation electrodes are applied, with the exception of off-pump coronary artery bypass (OPCAB) operations, as internal defibrillation with the internal shock paddles is difficult or impossible.

For central venous access, patients receive a 4-lumen central venous catheter, supplemented by a 9 FR venous catheter for volume access, typically inserted into the right internal jugular vein.

### 5.2. Transesophageal Echocardiography (TEE)

Comprehensive transesophageal echocardiography represents the gold standard for perioperative monitoring in cardiac surgery with some views being of central importance in minimally invasive cardiac surgery, given the potential contraindications [15]. Utilizing a comprehensive examination algorithm TEE facilitates assessment pre-, intra-, and postoperatively, enhancing surgical precision, confirming surgical indication, the positioning of bypass cannula, and patient safety.

### 5.3. Cerebral Oximetry

In the context of cardiac surgery, particularly in procedures with an elevated risk of cerebral ischemia, such as aortic surgery, which compromises the perfusion of the supra-aortic branches, near-infrared spectroscopy (NIRS) is a standard tool for the early detection of inadequate cerebral tissue oxygenation. It is essential to recognize that NIRS is associated with several inherent limitations. The optimal outcomes remain a topic of scientific investigation [16,17]. Cardiac surgery, particularly in procedures with heightened risks of cerebral ischemia, aortic surgeries affecting perfusion of supra-aortal branches, and near-infrared spectroscopy (NIRS) serve as vital tools for early detection of inadequate cerebral tissue oxygenation. Through continuous monitoring of cerebral oxygen saturation, surgical teams can promptly respond to potential complications, optimizing cerebral perfusion to mitigate the risk of neurological damage. Cerebral oximetry, an essential component of all cardiac procedures, ensures continuous, non-invasive assessment of cerebral blood flow dynamics. By discerning deviations indicative of compromised cerebral perfusion, cerebral oximetry informs management protocols outlined in a standardized approach. In particular, cerebral oximetry is advantageous in surgeries necessitating selective antegrade cerebral perfusion, such as aortic arch procedures, as it delivers indispensable insights into perfusion adequacy, enhancing surgical precision and patient safety.

### 5.4. Temperature Management

Temperature management is of paramount importance to coagulation and hemodynamic stability as well as in the context of ERACS [18]. The strategies employed are specifically adapted to align with the intended utilization of cardiopulmonary bypass (CPB) [19]. While the heart-lung machine can regulate patients’ temperature during on-pump surgeries, significant temperature loss can occur during off-pump procedures. Therefore, passive as well as proactive warming measures, including prewarmed blankets, warming mats, and infusion warmers, are initiated prior to anesthesia induction for off-pump surgeries in the absence of CPB. Heat mats used alone or in addition to heat blankets have resulted in sustained body temperature even during prolonged off-pump bypass surgeries. Consequently, heat mats are employed for all off-pump procedures, whereas CPB is utilized to regulate body temperature during on-pump procedures and warming blankets are utilized pre- and post-bypass. Core temperature monitoring via bladder catheterization ensures precise temperature control throughout the procedure.

### 5.5. Peripheral Cannulation

**Figure 1:**
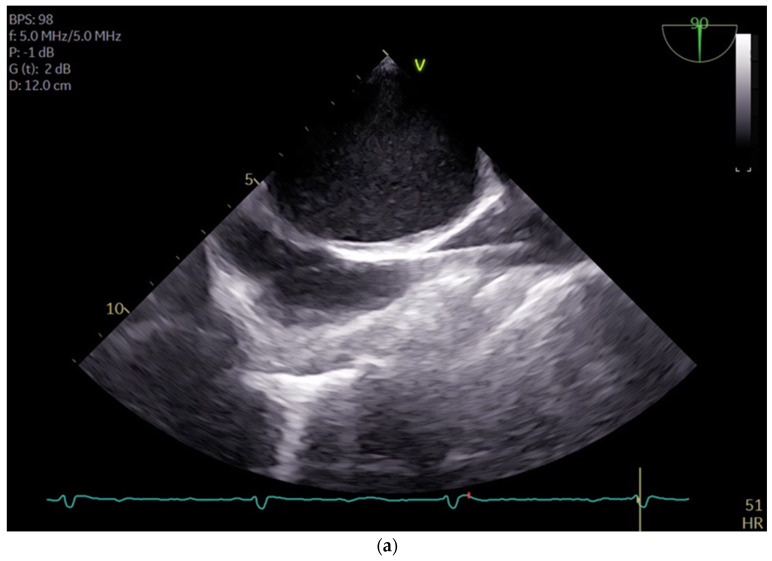

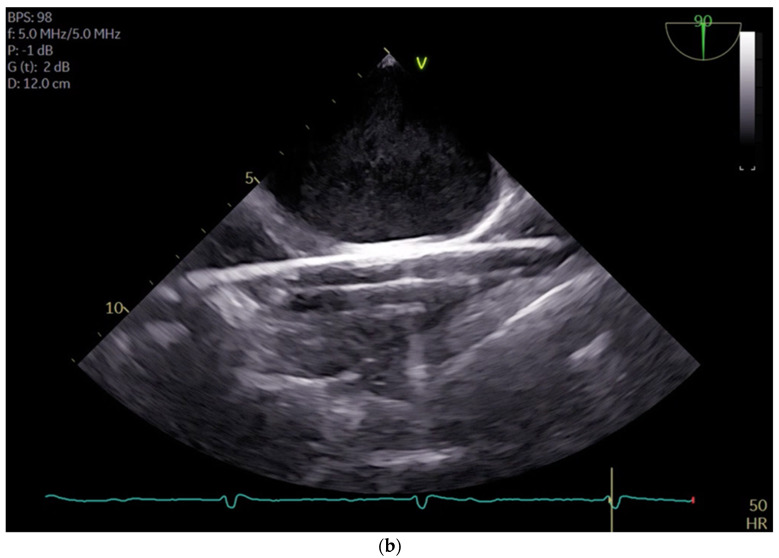
(**a**) shows the midesophageal bicaval view with the venous wire via the inferior vena cava into the superior vena cava; (**b**) shows the midesophageal bicaval view, with the cannula passing through the right atrium.

**Figure 2:**
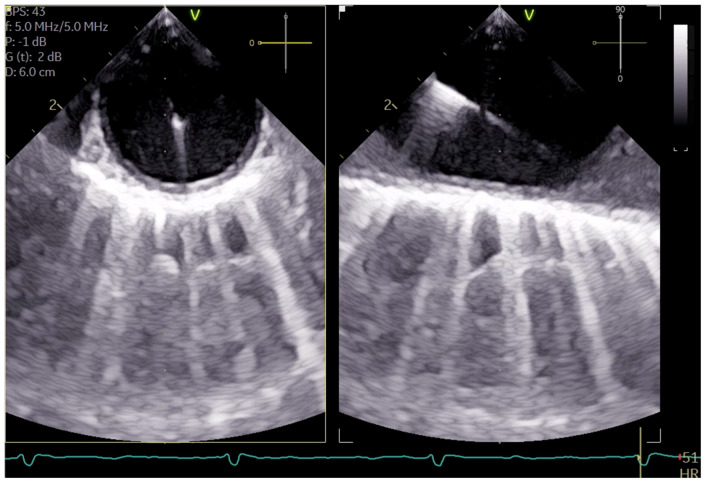
Shows the arterial wire in the descending aortic short-axis (SAX) and long-axis (LAX).

The cannulation of peripheral vessels for the purpose of establishing cardiopulmonary bypass (CPB) in minimal invasive cardiac surgery requires a meticulous approach. This is necessary in order to facilitate optimal redirection of blood flow. The femoral artery and vein, as well as the right internal jugular vein, are commonly employed vessels for cannulation [9]. These vessels offer accessible peripheral entry points for the insertion of cannulas, allowing for the efficient initiation of CPB while minimizing trauma to surrounding tissues [20]. Furthermore, advancements in surgical techniques have enabled the utilization of peripheral vessels such as the axillary artery, providing alternative cannulation sites that further enhance the minimally invasive nature of the procedure. This is achieved by potentially beneficial antegrade flow during cardiac procedures, which is currently under investigation [21]. By carefully selecting and cannulating these vessels, surgeons can effectively establish CPB with precision and safety, thereby facilitating successful outcomes in minimal invasive cardiac surgery. For femoral veins, a lengthy cannula (Bio-Medicus 23/25 FR multistage femoral venous cannula, Medtronic, Minneapolis, MN, USA) is introduced into the inferior vena cava, primarily via the right femoral vein, with echocardiographic guidance (refer to Videos S1 and S2). According to the established protocol, the guidewires are visualized. The venous wire and cannula are depicted through TEE in the midesophageal bicaval view (see Figure 1a,b). The arterial wire is visualized in the descending aortic short-axis (SAX) and long-axis (LAX) views (see Figure 2 and Video S3). The exclusion of malposition such as in the hepatic vein or interatrial septum perforation is carried out.

In certain cases, an additional cannulation of the superior vena cava (SVC) may be necessary, contingent on patient parameters such as body weight or specific surgical requirements, such as those pertaining to right atrial interventions. Indications for SVC cannulation include procedures requiring total bypass, such as tricuspid reconstruction/replacement, or procedures involving right atriotomy like ASD repair or mass resections for conditions such as tumors or thrombi. Furthermore, we perform SVC cannulation for all MICS procedures undergoing partial bypass in patients over 80 kg body weight for improved venous drainage.

**Figure 3:**
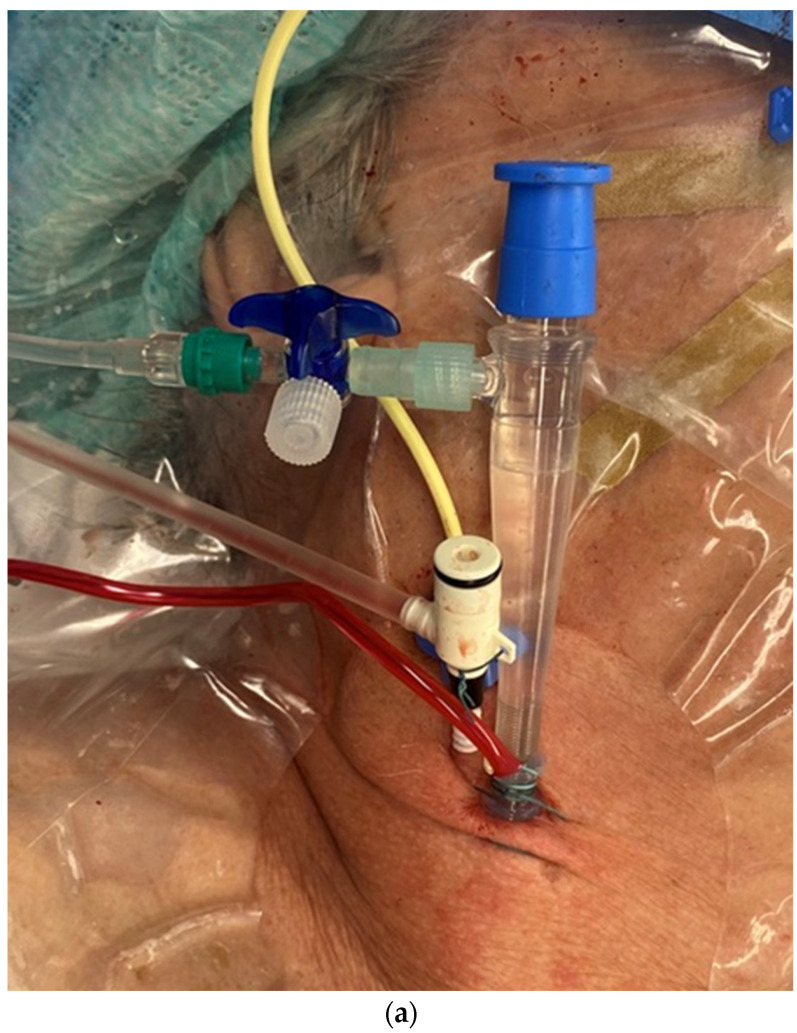

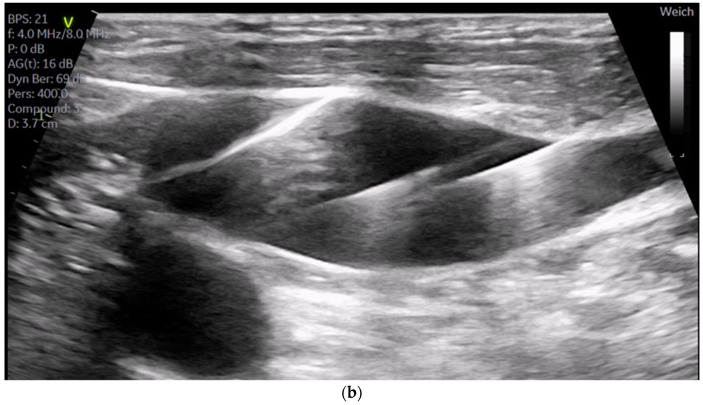
(**a**) shows the catheters inserted in the internal jugular vein right from cranial to caudal as follows: central 4-lumen central venous catheter, 1-lumen 9 FR catheter, and the CPB cannula; (**b**) shows three guidewires in the right internal jugular vein.

The SVC drainage cannula (Edwards Fr 16/18 OptiSite arterial cannula) is placed via the right jugular vein under ultrasound guidance simultaneously with the central venous catheter insertion. The cannula is positioned caudally to the central venous catheter and other venous lines (see Figure 3a,b). It is important to note that cannulation is performed above the superior thorax aperture to avoid potential complications resulting from intrathoracic vascular damage. We do not perform side-separated cannulation of the central venous catheter and CPB cannula at our center. This approach offers advantages in terms of time management and protection of the contralateral side for possible subsequent punctures during hospitalization. No adverse effects were observed in association with the multiple access procedure.

The positioning of the wires and cannulas is meticulously executed exclusively under ultrasound guidance, in accordance with the center’s policy and prevailing guidelines [22]. Prior to placement, the cannula is coated with 1 mL/2500 IU of heparin, with the objective of preventing immediate coagulation and clot formation subsequent to insertion. Following insertion into the internal jugular vein and after retrograde blood filling, the cannula is continuously flushed with a heparin-added full electrolyte solution (5000 IU heparin/500 mL) to sustain patency and prevent clot formation. A secure fixation is ensured with a pre-laid purse-string suture and a tourniquet is utilized to establish temporary fixation during the procedure. Post-completion of cardiopulmonary bypass (CPB) the remaining blood is flushed, the tourniquet is loosened, subsequently the cannula is carefully removed, and the incision site is closed using the pre-applied purse-string suture.

### 5.6. On-Table Extubation after Minimally Invasive Cardiac Surgery

Our anesthesiologic approach prioritizes on-table extubation (OTE) for MICS procedures in alignment with the Enhanced Recovery After Surgery (ERAS) concept [23,24,25]. Although randomized controlled trials on OTE are still pending, Jaquet et al. were able to demonstrate, in a retrospective analysis of 294 patients, that OTE was not associated with an increased incidence of respiratory complications and was associated with a lower risk of postoperative pneumonia and reduced requirement of vasopressors [19].

Preoperative screening allows for the identification of potential obstacles to on-table extubation. Subsequently, a comprehensive team evaluation precedes extubation. Extubation is initiated if the patient meets all criteria including adequate warmth, unobstructed gas exchange, competent coagulation, limited bleeding, and within-range vasopressor requirements. It is only performed if all team members agree at the end of the surgery. Weaning and extubation occur in the operating theater before patient transfer to the Intensive Care Unit (ICU).

### 5.7. MIDCAB

Minimally invasive direct coronary artery bypass graft (MIDCAB) surgery is a minimally invasive approach that is typically performed on the left anterior descending artery (LAD) at the front of the heart through a left-sided intercostal incision for left coronary artery access. However, this approach can also be utilized for the right coronary artery through a right-sided anterolateral minithoracotomy. Bypass grafting is performed on the beating heart and is typically employed for coronary grafts involving one or two vessels only. It is important to select the proper patients in order to achieve optimal results.

In order to ensure a direct and unrestricted view of the surgical field, OLV is employed for MIDCAB. This necessitates the use of a double-lumen tube for intubation, which enables lung isolation and selective ventilation. In accordance with our internal protocol, a bronchoscopy is conducted prior to skin incision to confirm the correct placement of the tube, with any necessary adjustments being made. As previously stated, a left-sided DTL is employed for all lung separations due to the reported inferior clinical performance of a right-sided DLT [12]. In cases where lung isolation is challenging, a bronchus blocker is utilized under bronchoscopic guidance as an alternative approach for lung isolation. While MIDCAB surgery is in general performed as an off-pump procedure at the beating heart it may occasionally be conducted utilizing mechanical support for patients with severely reduced ejection fraction. On this occasion peripheral cannulation is performed or a balloon pump is placed at the beginning of the surgery via femoral access. Temperature management is crucial and therefore warming is initiated prior to anesthesia induction using heating mats and blankets (Twinwarm BB and Universal III, MoeckWarmingSystems^®^ Hamburg, Germany).

### 5.8. OPCAB

Off-pump coronary artery bypass (OPCAB) surgery is performed in the presence of sternotomy, and therefore does not formally count as part of minimally invasive cardiac surgery according to the AHA definition. However, we present the special features for anesthesia here, as these can be challenging.

In this technique, the surgeon performs coronary artery bypass grafting without stopping the heart or utilizing a heart-lung bypass machine. This approach offers potential benefits, such as a reduced risk of complications associated with cardiopulmonary bypass, reduced recovery times, and potentially better outcomes for certain patients compared to on-pump surgery. By avoiding the use of the heart-lung machine, OPCAB minimizes the systemic inflammatory response associated with conventional bypass surgery and reduces the risk of bleeding, potentially leading to faster postoperative recovery. OPCAB is often used to achieve the goal of reducing trauma to the patient and improving overall surgical outcomes. In some cases, for patients with severely reduced ejection fraction OPCAB can be performed under mechanical protection using cardiopulmonary bypass, IABP, or impella (on-pump beating technique).

Monitoring, anesthesia induction, and airway management are analogous to those of on-pump surgery. The anesthesiologist must address the hemodynamic fluctuations induced by heart positioning necessary for bypass grafting. This involves preload and afterload adjustments and administering vasopressors as warranted.

Off-pump CABG presents anesthesiologists with unique challenges that require precise management of hemodynamic stability through vasopressor application and targeted fluid management. Therefore, continuous cardiac function monitoring, hemodynamics, and end-organ perfusion are essential for early detection and prompt management of any deviations from the desired physiological parameters. Close interaction between all perioperative team members is imperative to ensure the successful outcome of off-pump CABG procedures. Maintaining adequate cerebral perfusion pressure is crucial for the prevention of POCD and delirium. The utilization of the Hypotension Prediction Index (HPI) parameter, developed by Edwards Lifesciences (Irvine, CA, USA), has been demonstrated to result in a reduction in critically low mean arterial pressure (MAP). The HPI is an algorithm based on the characteristics of the arterial pressure curve and has been shown to possess a high degree of sensitivity and specificity in predicting hypotensive episodes during cardiac surgeries [26].

### 5.9. Aortic Valve Repair/Replacement

The most common approach for performing aortic valve surgery is via a right anterior mini-thoracotomy. The surgical approach involves a limited skin incision and access through the third intercostal space, utilizing a soft tissue retractor for optimal exposure while preserving the integrity of surrounding structures such as the right internal thoracic artery and vein. Our institution utilizes an endoscopic approach. A 3D camera and Chitwood clamp are placed via the second intercostal space, with careful opening of the pericardium above the phrenic nerve to facilitate valve exposure [27]. This approach is safe and achieves excellent results in high-volume centers [28]. At our institution the standard protocol for this MICS procedure involves intubation and ventilation via a single lumen tube and total bilateral lung deflation after the establishment of CPB. This approach does not result in any restriction of the surgical field of vision [27], nor does it increase the risk of reperfusion edema compared to OLV.

Transesophageal echocardiography (TEE) is employed to evaluate the aortic valve preoperatively, with regard to the pathology. This includes determination of the valve opening area via the continuity equation and measurement of the aortic annulus for decision of prosthesis size. A three-dimensional annulus size assessment may be more adequate than two-dimensional. The guidance of the surgeon for peripheral puncture and cannula positioning is provided via TEE in the midesophageal bicaval view and the views of the descending aorta (SAX and LAX) [29].

Prior to termination of the cardiopulmonary bypass (CPB) procedure, the surgical result is verified by means of transesophageal echocardiography (TEE). Following completion of CPB, a re-evaluation is performed with a focus on valve function, the presence of new onset stenosis or regurgitation, as well as the occurrence of new ventricular movement disorders. The aorta is evaluated to exclude any dissection following de-clamping and de-canulation.

### 5.10. Mitral Valve Repair/Replacement

Endoscopic minimally invasive cardiac surgery (MICS) for mitral valve repair involves a 3–5 cm skin incision or peri-areolar approach above the right fourth intercostal space, aided by a 3D camera, to facilitate exposure of the mitral valve through incision via the interatrial groove and left atriotomy. Minimally invasive mitral valve procedures are conducted with great precision under the guidance of basic cardioanesthesia monitoring and meticulous vascular access protocols. For patients with a body weight exceeding 80 kg, a jugular cannula is carefully inserted into the right internal jugular vein and advanced to the innominate vein. The cannula insertion process is guided by echocardiography, ensuring accuracy and safety. Our center does not execute OLV during valve procedures.

TEE is used to perform a comprehensive evaluation of the mitral valve, pertaining to the pathogenesis of regurgitation in general and measuring the length of the anterior mitral leaflet (AML) and the mitral annulus for selection of the annuloplasty ring. Additionally, the potential for systolic anterior motion (SAM) is assessed via TEE.

Occlusion of the circumflex artery is a rare but serious complication after mitral valve surgery. The patency of the circumflex artery and regional wall motion abnormalities are evaluated by TEE [30].

### 5.11. Tricuspid Valve Repair/Replacement

Minimally invasive cardiac surgery for the tricuspid valve is performed concomitantly with mitral valve surgery. A total bypass is required for interventions on the tricuspid valve. Bicaval venous cannulation is performed through the external jugular and femoral veins, while arterial cannulation is performed through the common femoral artery. For the total bypass, bulldog vascular clamps are used for the superior and inferior vena cava. During the total bypass, vasoactive drugs are administered via the heart-lung machine.

### 5.12. Mass Resection

The advantages of MICS can also be utilized in the removal of benign or malignant cardiac masses. The choice of cannulation strategy depends on the location of the tumor. Intraoperative TEE plays a key role in determining and verifying the surgical results after removal of the tumor.

### 5.13. Bleeding

Minimally invasive procedures inherently involve small incisions, reducing the likelihood of extensive bleeding. The routine administration of coagulation factors is not recommended for these cases. However, in the event of bleeding not attributed to the surgical site, a prompt thromboelastographic assessment is conducted, followed by targeted optimization of blood coagulation.

Although the optimal dosaging is still under investigation, tranexamic acid (TXA) is commonly used in cardiac surgery to reduce bleeding and the need for blood transfusions. At our institution tranexamic acid is administered as a standard protocol for all patients, with the exception of those undergoing MIDCAB surgery. A bolus dose of 10 mg/kg is initially administered, followed by a continuous infusion at 5 mg/kg/h. The anticoagulant effect of heparin is reversed with protamine, with real-time monitoring of activated clotting time (ACT) at the bedside to ensure optimal hemostasis.

### 5.14. Pain Management

At our center, we adopt a multifaceted approach to pain management. During surgery, either sufentanil or remifentanil is administered as opioids to effectively alleviate acute pain. The infiltration of the chest wall is carried out by the surgeon using a local anesthetic (10 mL 0.2% ropivacaine). Following surgery, peripheral analgesics (the first choice is metamizole (Novalgin^®^), or paracetamol as an alternative for metamizole allergy) are commonly employed to manage postoperative discomfort. Additionally, intravenous piritramide is available for pain relief as necessary. While regional anesthesia such as PECS II blockade is not routinely utilized, our comprehensive strategy is designed to minimize discomfort and facilitate early mobilization and recovery, ensuring optimal patient outcomes.

## 6. Conclusions

Minimal invasive cardiac surgeries have demonstrated excellent outcomes, with reduced morbidity, shorter recovery times, and improved patient satisfaction compared to traditional open-heart surgery at experienced centers. These results highlight the effectiveness and safety of MICS procedures in appropriately selected patients [31].

Anesthesia management is crucial for the outcome of minimal invasive cardiac surgery and requires careful consideration to ensure patient safety and optimal surgical conditions involving standardized treatment pathways. Overall, anesthesiologists must collaborate closely with the surgical team and perfusionists to address specific anesthesia implications related to cannulation strategies, hemodynamic management, lung isolation, temperature and fluid management, and coagulation control. Monitoring, including transesophageal echocardiography, is of great importance for the placement of catheters as well as for pre- and post-operative diagnostics. The management should be geared towards the earliest possible extubation with suitable patients.

## Supplementary Materials

The following supporting information can be downloaded at: https://www.mdpi.com/article/10.3390/jcm13133939/s1, Video S1: ME bicaval guidewire passing RA; Video S2: ME bicaval cannula passing RA; Video S3: desc. Ao. Guidewire.

## References

[1] Iyigun T., Kaya M., Gulbeyaz S.O., Fistikci N., Uyanik G., Yilmaz B., Onan B., Erkanli K. (2017). Patient body image, self-esteem, and cosmetic results of minimally invasive robotic cardiac surgery. Int. J. Surg..

[2] Parnell A., Prince M. (2018). Anaesthesia for minimally invasive cardiac surgery. BJA Educ..

[3] Aston D., Zeloof D., Falter F. (2023). Anaesthesia for Minimally Invasive Cardiac Surgery. J. Cardiovasc. Dev. Dis..

[4] Ilcheva L., Risteski P., Tudorache I., Haussler A., Papadopoulos N., Odavic D., Rodriguez Cetina Biefer H., Dzemali O. (2023). Beyond Conventional Operations: Embracing the Era of Contemporary Minimally Invasive Cardiac Surgery. J. Clin. Med..

[5] Glauber M., Ferrarini M., Miceli A. (2015). Minimally invasive aortic valve surgery: State of the art and future directions. Ann. Cardiothorac. Surg..

[6] Dieberg G., Smart N.A., King N. (2016). Minimally invasive cardiac surgery: A systematic review and meta-analysis. Int. J. Cardiol..

[7] Baishya J., George A., Krishnamoorthy J., Muniraju G., Chakravarthy M. (2017). Minimally invasive compared to conventional approach for coronary artery bypass grafting improves outcome. Ann. Card Anaesth..

[8] Goyal A., Chhabra L., Parekh A., Bhyan P., Khalid N. (2024). Minimally Invasive Aortic Valve Surgery. StatPearls.

[9] Lamelas J., Aberle C., Macias A.E., Alnajar A. (2020). Cannulation Strategies for Minimally Invasive Cardiac Surgery. Innovations.

[10] Ross A.F., Ueda K. (2010). Pulmonary hypertension in thoracic surgical patients. Curr. Opin. Anaesthesiol..

[11] Kim H.Y., Baek S.H., Je H.G., Kim T.K., Kim H.J., Ahn J.H., Park S.J. (2016). Comparison of the single-lumen endotracheal tube and double-lumen endobronchial tube used in minimally invasive cardiac surgery for the fast track protocol. J. Thorac. Dis..

[12] Kaplan T., Ekmekci P., Kazbek B.K., Ogan N., Alhan A., Kocer B., Han S., Tuzuner F. (2015). Endobronchial intubation in thoracic surgery: Which side should be preferred?. Asian Cardiovasc. Thorac. Ann..

[13] Irisawa Y., Hiraoka A., Totsugawa T., Chikazawa G., Nakajima K., Tamura K., Yoshitaka H., Sakaguchi T. (2016). Re-expansion pulmonary oedema after minimally invasive cardiac surgery with right mini-thoracotomy. Eur. J. Cardiothorac. Surg..

[14] Moss E., Halkos M.E., Binongo J.N., Murphy D.A. (2017). Prevention of Unilateral Pulmonary Edema Complicating Robotic Mitral Valve Operations. Ann. Thorac. Surg..

[15] Aybek T., Doss M., Abdel-Rahman U., Simon A., Miskovic A., Risteski P.S., Dogan S., Moritz A. (2005). Echocardiographic assessment in minimally invasive mitral valve surgery. Med. Sci. Monit..

[16] Chiong X.H., Wong Z.Z., Lim S.M., Ng T.Y., Ng K.T. (2022). The use of cerebral oximetry in cardiac surgery: A systematic review and meta-analysis of randomized controlled trials. Ann. Card Anaesth..

[17] Rogers C.A., Stoica S., Ellis L., Stokes E.A., Wordsworth S., Dabner L., Clayton G., Downes R., Nicholson E., Bennett S. (2017). Randomized trial of near-infrared spectroscopy for personalized optimization of cerebral tissue oxygenation during cardiac surgery. Br. J. Anaesth..

[18] Maj G., Regesta T., Campanella A., Cavozza C., Parodi G., Audo A. (2022). Optimal Management of Patients Treated With Minimally Invasive Cardiac Surgery in the Era of Enhanced Recovery After Surgery and Fast-Track Protocols: A Narrative Review. J. Cardiothorac. Vasc. Anesth..

[19] Jaquet O., Gos L., Amabili P., Donneau A.F., Mendes M.A., Bonhomme V., Tchana-Sato V., Hans G.A. (2023). On-table Extubation After Minimally Invasive Cardiac Surgery: A Retrospective Observational Pilot Study. J. Cardiothorac Vasc. Anesth..

[20] Pozzoli A., Torre T., Toto F., Theologou T., Ferrari E., Demertzis S. (2022). Percutaneous Venous Cannulation for Minimally Invasive Cardiac Surgery: The Safest and Effective Technique Described Step-by-Step. Front. Surg..

[21] Kruse J., Silaschi M., Velten M., Wittmann M., Alaj E., Ahmad A.E., Zimmer S., Borger M.A., Bakhtiary F. (2023). Femoral or Axillary Cannulation for Extracorporeal Circulation during Minimally Invasive Heart Valve Surgery (FAMI): Protocol for a Multi-Center Prospective Randomized Trial. J. Clin. Med..

[22] Apfelbaum J.L., Rupp S.M., Tung A., Connis R.T., Domino K.B., Grant M.D., Mark J.B. (2020). Practice Guidelines for Central Venous Access 2020: An Updated Report by the American Society of Anesthesiologists Task Force on Central Venous Access. Anesthesiology.

[23] Salenger R., Morton-Bailey V., Grant M., Gregory A., Williams J.B., Engelman D.T. (2020). Cardiac Enhanced Recovery After Surgery: A Guide to Team Building and Successful Implementation. Semin. Thorac. Cardiovasc. Surg..

[24] Ender J., Borger M.A., Scholz M., Funkat A.K., Anwar N., Sommer M., Mohr F.W., Fassl J. (2008). Cardiac surgery fast-track treatment in a postanesthetic care unit: Six-month results of the Leipzig fast-track concept. Anesthesiology.

[25] Zakhary W.Z.A., Turton E.W., Flo Forner A., von Aspern K., Borger M.A., Ender J.K. (2019). A comparison of sufentanil vs. remifentanil in fast-track cardiac surgery patients. Anaesthesia.

[26] Shin B., Maler S.A., Reddy K., Fleming N.W. (2021). Use of the Hypotension Prediction Index During Cardiac Surgery. J. Cardiothorac. Vasc. Anesth..

[27] El-Sayed Ahmad A., Salamate S., Bakhtiary F. (2022). Lessons learned from 10 years of experience with minimally invasive cardiac surgery. Front. Cardiovasc. Med..

[28] Bakhtiary F., El-Sayed Ahmad A., Amer M., Salamate S., Sirat S., Borger M.A. (2021). Video-Assisted Minimally Invasive Aortic Valve Replacement Through Right Anterior Minithoracotomy for All Comers With Aortic Valve Disease. Innovations.

[29] Kasel A.M., Cassese S., Bleiziffer S., Amaki M., Hahn R.T., Kastrati A., Sengupta P.P. (2013). Standardized imaging for aortic annular sizing: Implications for transcatheter valve selection. JACC Cardiovasc. Imaging.

[30] Ender J., Selbach M., Borger M.A., Krohmer E., Falk V., Kaisers U.X., Mohr F.W., Mukherjee C. (2010). Echocardiographic identification of iatrogenic injury of the circumflex artery during minimally invasive mitral valve repair. Ann. Thorac. Surg..

[31] Monsefi N., Alaj E., Sirat S., Bakhtiary F. (2022). Postoperative results of minimally invasive direct coronary artery bypass procedure in 234 patients. Front. Cardiovasc. Med..

